# SJB2-043, a USP1 Inhibitor, Suppresses A549 Cell Proliferation, Migration, and EMT via Modulation of PI3K/AKT/mTOR, MAPK, and Wnt Signaling Pathways

**DOI:** 10.3390/cimb47030155

**Published:** 2025-02-27

**Authors:** Lipeng Wu, Meng Yu, Huosheng Liang, Long Lin, Huajian Li, Guangyang Chen, Halimulati Muhetaer, Jingjing Li, Bo Wu, Xuejing Jia, Yuanye Dang, Guodong Zheng, Chuwen Li

**Affiliations:** 1 Guangzhou Municipal and Guangdong Provincial Key Laboratory of Molecular Target & Clinical Pharmacology, NMPA and State Key Laboratory of Respiratory Disease, School of Pharmaceutical Sciences and Affiliated Traditional Chinese Medicine Hospital, Guangzhou Medical University, Guangzhou 510645, China; 2 School of Biomedical and Pharmaceutical Sciences, Guangdong University of Technology, Guangzhou 510006, China; 3 Department of Rehabilitation Sciences, Faculty of Health and Social Sciences, Hong Kong Polytechnic University, Hong Kong 999077, China; 4 College of Food Science and Technology, Guangdong Ocean University, Zhanjiang 524088, China

**Keywords:** epithelial–mesenchymal transition (EMT), non-small cell lung cancer (NSCLC), USP1 inhibitor, SJB2-043

## Abstract

Objective: Non-small cell lung cancer (NSCLC) remains one of the most significant contributors to cancer-related mortality. This investigation explores the influence and underlying mechanisms of the USP1 inhibitor SJB2-043 on A549 cells, with the aim of advancing the development of anti-NSCLC therapeutics. Methods: Publicly available databases were utilized to assess USP1 expression and its association with the progression of NSCLC. Gene expression variations were ascertained through RNA sequencing, followed by the Kyoto Encyclopedia of Genes and Genomes and Gene Ontology pathway enrichment evaluations. Various doses of SJB2-043 were administered to A549 cells to evaluate its impact on cell multiplication, motility, apoptosis, and the cell cycle using CCK-8 assays, colony formation, wound healing, flow cytometry, and Western blotting (WB). Results: USP1 was found to be overexpressed in NSCLC specimens and linked to adverse prognosis. Treatment with SJB2-043 markedly inhibited A549 cell proliferation and migration, diminished clonogenic potential, and triggered apoptosis in a dose-dependent manner. Modifications in the cell cycle were observed, showing an elevated percentage of cells in the G2 phase while exhibiting a parallel decline in the G_1_ phase. WB examination demonstrated diminished protein levels of N-cadherin, CyclinB_1_, CDK1, C-myc, Bcl-2, p-ERK/ERK, p-p38/p38, p-JNK/JNK, p-AKT/AKT, and p-mTOR/mTOR, alongside an upregulation of E-cadherin, ZO-1, occludin, p53, Bax, p-β-catenin/β-catenin, and GSK3β. Conclusions: SJB2-043 exerts a suppressive effect on A549 cell proliferation, migration, and epithelial–mesenchymal transition while enhancing apoptosis. These cellular effects appear to be mediated through the inhibition of the MAPK, Wnt/β-catenin, and PI3K/AKT/mTOR signaling cascades, in addition to modulation of the cell cycle.

## 1. Introduction

Lung cancer (LC) represents the predominant malignancy affecting the respiratory system and remains the leading contributor to cancer-related morbidity and mortality worldwide. In 2022, approximately 2.5 million new LC cases and around 1.8 million deaths were documented worldwide, emphasizing its profound impact on public health [1,2]. Non-small cell lung cancer (NSCLC) represents 80–85% of total lung cancer diagnoses, with a substantial proportion of cases identified at later stages, thus forfeiting the possibility of surgical intervention [3,4]. Genetic mutations, such as those in EGFR, KRAS, ALK, ROS1, and BRAF, within NSCLC cells, along with the dysregulated activation of pivotal signaling pathways encompassing PI3K/AKT/mTOR, MAPK, and Wnt cascades, propel tumor progression via multiple mechanisms [5,6]. These oncogenic alterations act synergistically to impair therapeutic responsiveness, enhance proliferative and metastatic capabilities, and collectively pose significant clinical hurdles in the management of NSCLC by promoting treatment resistance and disease aggressiveness [7]. These challenges emphasize the urgent necessity to discover novel therapeutic targets and devise efficacious therapeutic agents for the treatment of NSCLC.

Deubiquitinating enzymes (DUBs) have recently been identified as promising therapeutic targets in cancer research [8]. DUBs regulate protein stability, cellular signaling, and gene expression by removing ubiquitin from target proteins, thereby influencing crucial physiological processes such as tumor cell proliferation, survival, and migration [9]. Among these, ubiquitin-specific peptidase 1 (USP1) has garnered considerable interest because of its role in tumor development [10,11]. Research has demonstrated that USP1 stabilizes estrogen receptors in breast cancer, thereby promoting tumor growth [12] and inhibiting differentiation in osteosarcoma [13]. In NSCLC, the inhibition of USP1 has been shown to potentially reverse cisplatin resistance in preclinical models [14]. These collective studies indicate that USP1 holds potential as a promising target for cancer therapy [15].

Several USP1 inhibitors have been developed in recent years, demonstrating potential as anti-cancer agents. For instance, KSQ-4279 has been recognized as a promising therapy for overcoming poly(ADP-ribose) polymerase (PARP) inhibitor resistance in breast cancer susceptibility gene (*BRCA*)-mutant tumors, while ML323 demonstrates effectiveness in suppressing ovarian cancer cell proliferation by targeting USP1-regulated cell cycles [16]. SJB2-043, first synthesized and reported in 2013, has been demonstrated to inhibit USP1 function, exhibiting cytotoxic effects on leukemic cells [17]. Additionally, SJB2-043 has been found to impede the maintenance of cancer stem cells and DNA damage repair by modulating USP1 activity [18]. However, its therapeutic potential in NSCLC has yet to be fully explored.

This investigation examines the regulatory mechanisms of SJB2-043 and its effects on NSCLC cell multiplication and infiltration. By elucidating the molecular pathways modulated by SJB2-043, the aim is to advance the understanding of NSCLC pathogenesis and establish a theoretical foundation for the development of novel clinical therapeutics.

## 2. Materials and Methods

### 2.1. Drug

SJB2-043 (MedChemExpress, Monmouth Junction, NJ, USA).

### 2.2. Cell Culture

A549 cells were purchased from ATCC and cultured in RPMI-1640 medium (Gibco, San Francisco, CA, USA) comprising 10% fetal bovine serum (Vivacell, Shanghai, China) and 1% penicillin–streptomycin (Gibco, San Francisco, CA, USA) and kept at 37 °C in a 5% CO_2_ atmosphere with routine passaging.

### 2.3. Correlation Analysis Between USP1 and NSCLC Prognosis

The association between USP1 mRNA expression and markers linked to cell proliferation (Ki-67 and PCNA), anti-apoptosis (Bcl2), and epithelial–mesenchymal transition (EMT) (N-cadherin) was investigated through the use of lung adenocarcinoma data sourced from the UCSX Xena database (https://xena.ucsc.edu/, accessed on 2 November 2024). The quantities of USP1 protein present in USP1 in both tumor and normal tissues were assessed utilizing the human protein atlas (HPA) database 24.0 (https://www.proteinatlas.org/, accessed on 7 November 2024), while the UALCAN database (https://ualcan.path.uab.edu/analysis.html, accessed on 18 November 2024) was employed to explore the link between USP1 levels and NSCLC outcomes. (All the keywords were USP1 and lung adenocarcinoma).

### 2.4. RNA Sequencing, Differential Expression Analysis, and Kyoto Encyclopedia of Genes and Genomes (KEGG)

A549 cells were categorized into a control cohort and a drug-treated cohort (10 μM). Following 24 h of treatment, RNA-sequencing (RNA-seq) was conducted. Total RNA was procured from the tissues with the TRIzol reagent per the supplier’s protocol. Library preparation and mRNA sequencing were carried out by the Meiji Gene Technology Company. mRNA was enriched through the use of Oligo (dT) beads and subsequently fragmented. Reverse transcription was executed with random N6 primers to generate double-stranded DNA, and SOAPnuke 2.1.8 software executed quality control analysis on the procured sequencing data. Reference genome alignment was performed using HISAT v2.2.1 software, which relies on the Burrows–Wheeler Transform and Ferragina–Manzini method. Differential gene expression analysis was executed utilizing the “DESeq2” package, employing thresholds of FDR < 0.05 and |log2-fold change| > 1. To investigate the pharmacological mechanism of SJB2-043 in NSCLC, common differential genes were identified by intersecting the gene sets derived from the control cohort versus low-dose and control cohort versus high-dose comparisons. KEGG and Gene Ontology (GO) enrichment analyses of these common genes was conducted using the Metascape database, with enrichment thresholds set at *p* < 0.01 and a minimum overlap of 5.

### 2.5. CCK-8 Assay for Cell Viability

Cells in the logarithmic growth phase were harvested and placed into 96-well plates at 5000 cells per well. Upon completion of 24 h incubation, the drugs (0, 0.39, 0.156, 0.625, 2.5, 10 μM) were introduced, succeeded by an additional 24-h culture. Subsequently, a 10% CCK-8 reagent was introduced (Beyotime, Shanghai, China), and the plates underwent a 2-h incubation. The absorbance of each cohort was ascertained at 450 nm wavelength utilizing a microplate reader (BioTek epoch, Winooski, VT, USA).

### 2.6. Colony Formation (CF) Assay

A549 cells in the logarithmic growth phase were placed into 6-well plates at 1000 cells per well. Once the cells had adhered, they were treated with 2.5 μM and 10 μM drugs, respectively, with a blank control cohort included. The cells were kept for 10 days, with the drugs being replaced every 3 days. Upon completion of the culture period, the cells underwent PBS washing, fixation with 4% paraformaldehyde for 30 min, and crystal violet staining for 10 min, succeeded by PBS rinsing. After drying, the outcomes were analyzed utilizing ImageJ 1.54d software.

### 2.7. Flow Cytometry (FCM) Analysis of Cell Cycle with Propidium Iodide (PI) Staining

A549 cells from both the control and drug-treated cohorts (0.625, 2.5, 10 μM) were harvested and fixed in pre-cooled 75% ethanol for a duration of 4 h. Following centrifugation at 4 °C, the cells were resuspended in phosphate buffered saline (PBS) and underwent a second centrifugation, and the supernatant was discarded. The cells were then stained with 0.5 mL of PI staining solution (Beyotime, Shanghai, China) at 37 °C in the dark for 30 min. Data were subsequently acquired via flow cytometry (Beckman Coulter, Brea, CA, USA) and evaluated utilizing FlowJo software 10.8.1 (Tree Star, Mountain View, CA, USA).

### 2.8. Detection of Cell Apoptosis Utilizing Annexin V-FITC Apoptosis Kit

A549 cells from both the control and drug-treated cohorts (0.625, 2.5, 10 μM) were harvested and resuspended in PBS. Following this, 195 μL of loading buffer, 10 μL of PI, and 5 μL of FITC were introduced (Beyotime, Shanghai, China), and the cells underwent staining at ambient conditions for 10 min. Data were subsequently acquired via flow cytometry and evaluated utilizing FlowJo software 10.8.1 (Tree Star, Mountain View, CA, USA).

### 2.9. Wound-Healing Assay

A wound-healing assay was executed in 6-well plates. Parallel lines were marked on the reverse sides of the plates, and the cells were seeded at 6 × 10^5^ cells per well. Upon reaching 90% cell density, a scratch was made perpendicular to the lines utilizing a 200 μL pipette tip. The cells were subsequently rinsed with PBS three times and treated with 2.5 μM and 10 μM drug concentrations. Images were captured at 0, 12, 24, and 48 h, and the results were analyzed using ImageJ 1.54d software.

### 2.10. WB Analysis

After treating A549 cells with SJB2-043, RIPA lysis buffer (Beyotime, Shanghai, China) was added to extract protein from the cells. The extracted proteins were quantified (Thermo Fisher Scientific, Waltham, MA, USA), subjected to electrophoresis, and subsequently moved onto a polyvinylidene fluoride membrane (Merck, Hessen, Germany) via wet transfer. A 5% skim milk solution (Fude Biotechnology, Hangzhou, China) blocked the membranes for 1 h, after which primary antibodies were introduced and kept overnight at 4 °C with shaking. The membranes underwent triple washing (5 min each), HRP-conjugated secondary antibodies (Proteintech, Wuhan, China) were applied, and the membrane was incubated on a shaker at ambient conditions for 1 h. Following another three washes (5 min each), an enhanced chemiluminescence luminescent substrate (NCM Biotech, Shanghai, China) was applied to visualize the proteins. The results were then analyzed using a gel imaging system (Bio-Rad, Hercules, CA, USA). The primary antibodies used in this study included N-cadherin, GAPDH, β-tubulin, β-actin, p-38, p-p38, JNK, p-JNK, AKT, p-AKT (Affinity, Cincinnati, OH, USA), and E-cadherin, p-β-catenin, β-catenin, GSK3β, CDK1, CyclinB_1_, C-myc, β-actin, ERK, p-ERK, mTOR, and p-mTOR (Proteintech, Wuhan, China).

### 2.11. Statistical Analysis

Statistical analyses were conducted utilizing GraphPad Prism 9 (v9.0.1, GraphPad Software Inc., San Diego, CA, USA) with rigorous adherence to parametric test assumptions. Quantitative variables were reported as arithmetic mean ± standard deviation following normality confirmation. For dual-group comparisons, independent samples were evaluated using Student’s *t*-test. Multigroup analyses employed one-way ANOVA with Tukey’s post hoc correction. *p* values < 0.05 were considered as significant differences.

## 3. Results

### 3.1. Identification of USP1 as an Oncogene in NSCLC

**Figure 1:**
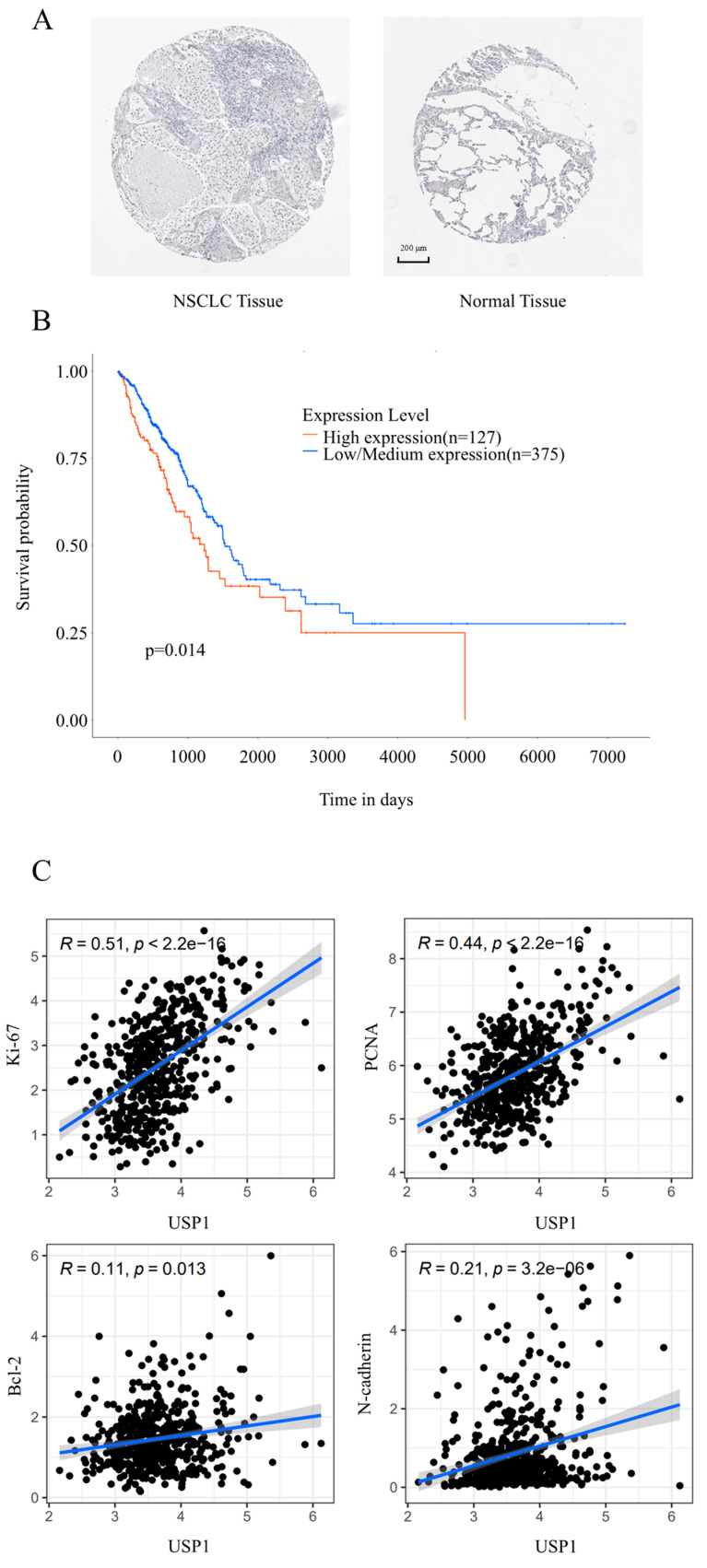
Protein expression of USP1 in human NSCLC tissues and normal tissues from the HPA database (**A**) and Bioinformatics analysis of USP1 in NSCLC. UALCAN database analysis of USP1 expression in relation to NSCLC prognosis (**B**). Correlation analysis of USP1 with Ki-67, PCNA, Bcl-2, and N-Cadherin mRNA expression in human lung adenocarcinoma tissues in the TCGA cohort (**C**). The following criteria were applied: *R* > 0.3: Moderate-to-strong correlation; *R* = 0.1–0.3: Weak correlation; *R* < 0.1: Negligible correlation; A *p* value < 0.05 was considered statistically significant.

Interrogation of the Human Protein Atlas (HPA) revealed differential overexpression of USP1 protein in lung adenocarcinoma specimens compared to adjacent normal parenchyma (Figure 1A), establishing its tumor-specific expression pattern. Furthermore, clinico-genomic integration demonstrated that USP1 hyperexpression correlates with adverse clinical outcomes (Figure 1B). To dissect USP1’s functional interactome, we employed Pearson correlation analysis [19] to assess the relationship between USP1 and proliferation/apoptosis and migration-related markers (Ki-67, PCNA, Bcl-2, N-cadherin) using mRNA expression data from TCGA lung adenocarcinoma samples. The results revealed a strong transcriptional covariation between USP1 and mitotic regulators Ki-67 and PCNA, positioning USP1 as a master regulator of cell progression, consistent with its known role in DNA repair [20] and cell cycle regulation [21]. Although the correlations with Bcl-2 and N-cadherin are relatively weak, their statistical significance (*p* < 0.05) indicates that these associations are not random. The large sample size of the TCGA dataset (*n* = 510) enhances our ability to detect even minor correlations, which may still be biologically meaningful. Furthermore, the functional experiments conducted in subsequent sections provide crucial support for these findings. Collectively, these results emphasize the clinical importance of USP1 in NSCLC and highlight its potential as a therapeutic target for the disease.

### 3.2. RNA-Seq and Bioinformatics Analysis of SJB2-043

**Figure 2:**
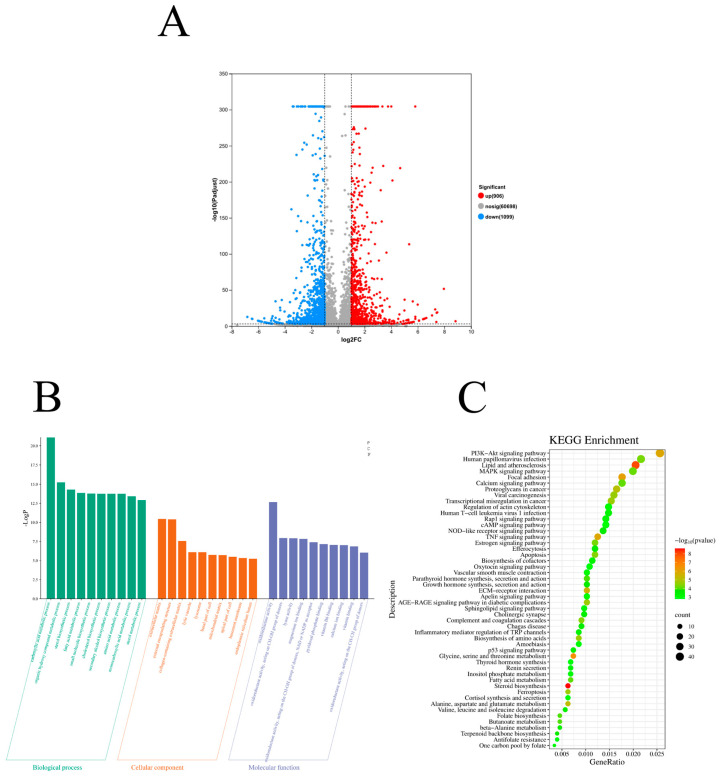
Volcano plot (**A**) and KEGG pathway (**B**) and GO enrichment analyses (**C**) of DEGs. The volcano plot demonstrates the effect of the experimental treatment on gene expression in A549 cells. The *X*-axis displays the log2 fold change of gene expression, while the *Y*-axis denotes the statistical significance of differential expression, represented by the −log10 *p*-value. Genes with significant upregulation are denoted by red dots (log2-fold change > 1, *p*-value < 0.05), while blue dots denote markedly downregulated genes (log2-fold change < −1, *p*-value < 0.05). Gray dots denote genes exhibiting no substantial changes.

To investigate the molecular mechanisms responsible for the suppression of NSCLC proliferation and migration by SJB2-043, RNA-seq is performed on A549 cells treated with SJB2-043 for 24 h. The analysis revealed the identification of 2005 differentially expressed genes (DEGs) after 24 h of treatment, with 906 genes showing upregulation and 1099 genes exhibiting downregulation (Figure 2A). The 2005 DEGs were subjected to KEGG and GO enrichment analysis using the Metascape database [22]. KEGG pathway enrichment evaluation suggested that these DEGs are predominantly associated with cancer-related signaling cascades, encompassing the PI3K/AKT pathway, MAPK pathway, and p53 signaling pathway (Figure 2B,C).

### 3.3. SJB2-043 Inhibits A549 Cell Proliferation and Inhibits the Clonogenic Ability of A549 Cells

**Figure 3:**
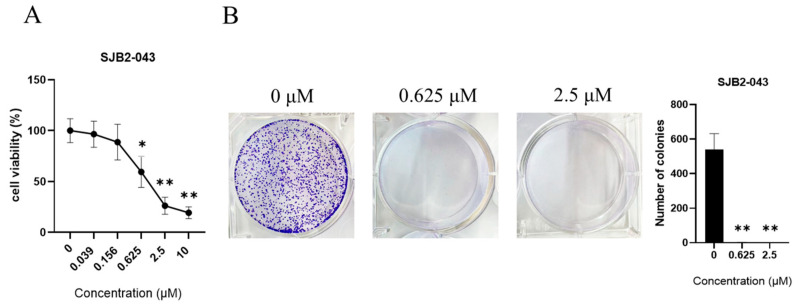
Effect of SJB2-043 on cell viability (**A**) and CF (**B**) of A549. A549 cells underwent treatment with diverse doses of SJB2-043 over 24 h. Cell viability measurements were executed utilizing the CCK-8 method. Data are denoted as the mean ± SD (*n* = 9). One-way ANOVA was employed to examine statistical significance. * *p* < 0.05, ** *p* < 0.01, compared with the control group. Representative images of CF in A549 cells exposed to varying levels of SJB2-043 for 10 days. Data are denoted as mean ± SD (*n* = 9). Statistical significance was ascertained utilizing one-way ANOVA. ** *p* < 0.01, compared with the control group.

SJB2-043’s cytotoxicity in A549 cells was assessed by exposing growing cells to multiple doses of the compound. CCK-8 assays demonstrated that SJB2-043 diminished cell viability in a dose-dependent fashion (Figure 3A). A CF assay was employed to assess the impact of SJB2-043 on cell growth and CF (Figure 3B). The findings indicated that SJB2-043 markedly suppressed the CF capacity of A549 cells.

Additionally, we conducted an experiment where normal human lung epithelial cells (Beas2B) were exposed to SJB2-043, and the outcomes revealed that even at a high concentration of 10 µM, SJB2-043 exerted minimal to no effect on Beas2B cells. This result underscores the remarkable safety profile of SJB2-043 for in vitro use (Figure S1).

### 3.4. SJB2-043 Promotes Apoptosis in A549 Cells

**Figure 4:**
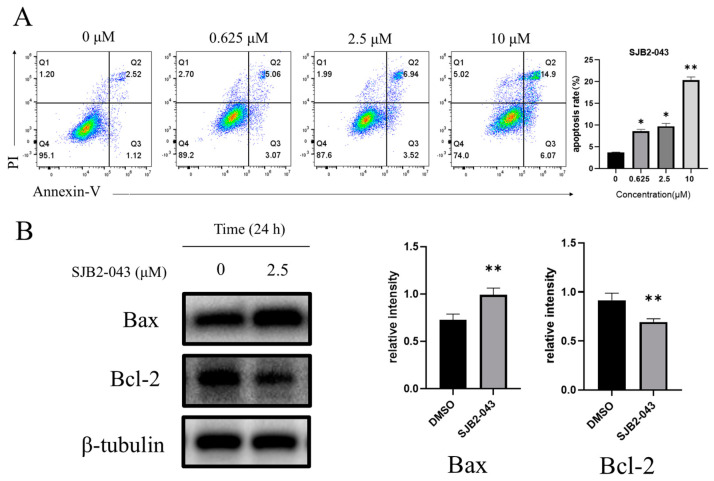
Effect of SJB2-043 on apoptosis of A549 cells. Apoptosis in A549 cells exposed to diverse doses of SJB2-043 for 24 h was evaluated by FCM (**A**). Data are denoted as mean ± SD (*n* = 9). Statistical significance was ascertained via one-way ANOVA. * *p* < 0.05, ** *p* < 0.01, compared with the control group. WB analysis of apoptosis marker expression level (Bax, Bcl-2) in A549 cells exposed to 2.5 μM SJB2-043 for 24 h (**B**). β-tubulin served as a loading control. Data are denoted as mean ± SD (*n* = 3). Statistical significance was examined utilizing Student’s *t*-test. ** *p* < 0.01, compared with the control group.

In this investigation, FCM coupled with Annexin V-FITC/PI double-staining was utilized to assess the proportion of apoptotic cells. The data revealed a marked elevation in both initial and advanced apoptotic cell populations with escalating concentrations of SJB2-043 (Figure 4A). To investigate the molecular impact of SJB2-043 on apoptosis, WB analysis was conducted to evaluate alterations in apoptosis-associated protein levels in SJB2-043-treated cells. After treatment, notable upregulation of Bax and downregulation of Bcl-2 expression was observed (Figure 4B). These results imply that SJB2-043 promotes apoptosis in A549 cells.

### 3.5. SJB2-043 Arrests A549 Cells in the G2 Phase

**Figure 5:**
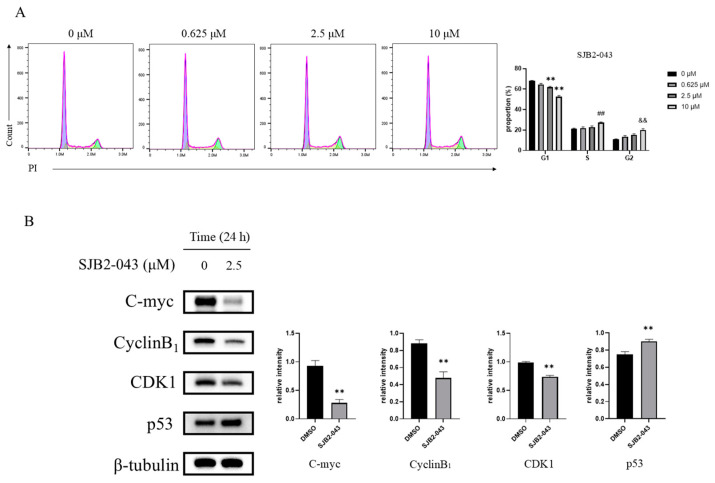
Effect of SJB2-043 on cell cycle in A549 cells. FCM was employed to assess the distribution of cell cycle phases in A549 cells exposed to diverse SJB2-043 concentrations over 24 h (**A**). The G1 phase is represented by the purple section, the S phase by the yellow section, and the G2 phase by the green section. Data are denoted as mean ± SD (*n* = 9). Statistical significance was ascertained utilizing one-way ANOVA. ** indicates *p* < 0.01 for the G1 phase compared to the control group, ## indicates *p* < 0.01 for the S phase compared to the control group, and && indicates *p* < 0.01 for the G2 phase compared to the control group. Subsequently, WB evaluation measured the abundance of essential G2 phase proteins (C-myc, CyclinB_1_, CDK1, p53) in A549 cells subjected to 2.5 μM SJB2-043 treatment for 24 h (**B**). β-tubulin functioned as the loading reference. Data are denoted as mean ± SD (*n* = 3), with statistical significance evaluated utilizing Student’s *t*-test. ** *p* < 0.01, compared with the control group.

Cell mitosis, fundamentally linked to cell multiplication, emerged as the key biological mechanism for examination. To examine the impact of SJB2-043 on mitosis, the cell cycle was analyzed utilizing FCM following PI staining. A549 cells underwent exposure to varying doses of SJB2-043 (0, 0.625, 2.5, 10 μM) for 24 h, and cell cycle progression was subsequently evaluated. As depicted in Figure 5A, treatment with SJB2-043 led to a significant elevation in the G2 phase cell population.

WB was then employed to evaluate alterations in key cell cycle-related protein level. With elevated doses of SJB2-043, upregulation of p53 protein expression was observed, while the expression levels of C-myc, CyclinB_1_, and CDK1 were markedly diminished (Figure 5B).

### 3.6. SJB2-043 Inhibits the Migration Capability of A549 Cells

**Figure 6:**
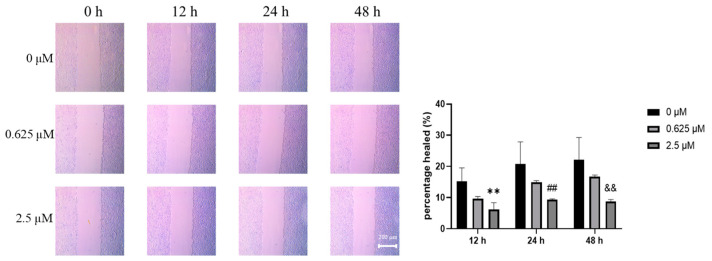
SJB2-043 suppresses the migration of NSCLC cells in a wound-healing assay. Representative images of wound healing in A549 cells exposed to varying concentrations of SJB2-043 at 0, 12, 24, and 48 h are presented. Data are denoted as mean ± SD (*n* = 9). Statistical significance was ascertained utilizing one-way ANOVA. ** represents *p* < 0.01 compared to the control group at the 12 h time point, ## represents *p* < 0.01 compared to the control group at the 24 h time point, && represents *p* < 0.01 compared to the control group at the 48 h time point.

As cancer cell migration serves a pivotal function in tumor metastasis and patient mortality, the effects of SJB2-043 on cancer cell migration were investigated. The wound-healing assay demonstrated that SJB2-043 markedly inhibited A549 cell migration in a dose-dependent manner (Figure 6).

### 3.7. SJB2-043 Downregulates N-Cadherin, but Upregulates the Epithelial Marker E-Cadherin, ZO-1, and Occludin in A549 Cells

**Figure 7:**
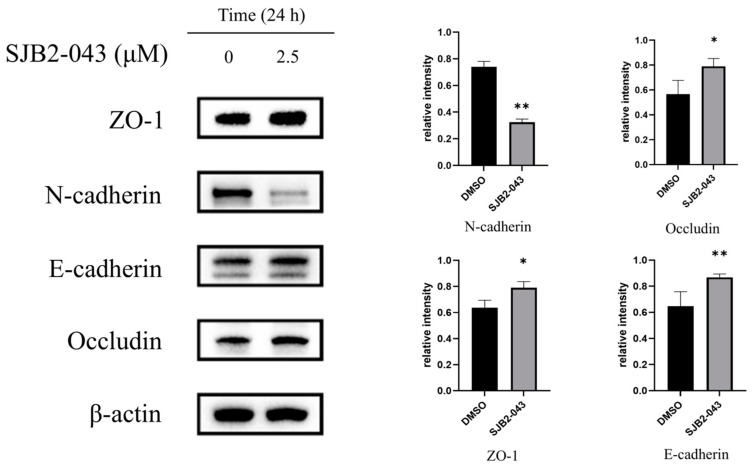
Effects of SJB2-043 on EMT protein expression in A549 cells. WB analysis was executed to evaluate the protein levels of EMT indicators (E-cadherin, N-cadherin, occludin, ZO-1) in A549 cells exposed to SJB2-043 at 2.5 µM for 1 day. β-actin was utilized as a loading control. Data are denoted as mean ± SD (*n* = 3). Statistical significance was ascertained utilizing Student’s *t*-test. * *p* < 0.05, ** *p* < 0.01, compared with the control group.

Wound-healing assays demonstrated that SJB2-043 markedly diminished the migratory capacity of A549 cells. Emerging oncobiology paradigms position EMT as a molecular linchpin governing NSCLC metastatic competence. Tumor specimens frequently demonstrate upregulated expression of mesenchymal markers N-cadherin, which have been clinically associated with advanced disease stages, diminished overall survival, and enhanced invasive capacity [23,24]. Conversely, epithelial markers E-cadherin, occluding, and ZO-1 exhibit significant downregulation in metastatic NSCLC lesions. Mechanistically, E-cadherin restoration suppresses metastatic dissemination through transcriptional repression of N-cadherin via ZEB1/2-mediated pathways, while ZO-1 and occludin reinforce intercellular tight junctions to impede tumor cell intravasation [25,26,27]. These reciprocal regulatory axes position EMT dynamics as critical determinants of NSCLC progression, serving as potential prognostic biomarkers and therapeutic targets against metastasis. Therefore, we conducted WB analysis, and further confirmed the regulatory effect of SJB2-043 on ZO-1, occludin, E-cadherin, and N-cadherin protein levels in A549 cells. It can be clearly observed that after treatment with SJB2-043, the expression of ZO-1 and E-cadherin is significantly upregulated, while the expression of vimentin and N-cadherin is suppressed. Therefore, we can conclude that SJB2-043 inhibits the migration ability of A549 cells by suppressing the EMT (Figure 7).

### 3.8. SJB2-043 Downregulates the MAPK Pathway in A549 Cells

**Figure 8:**
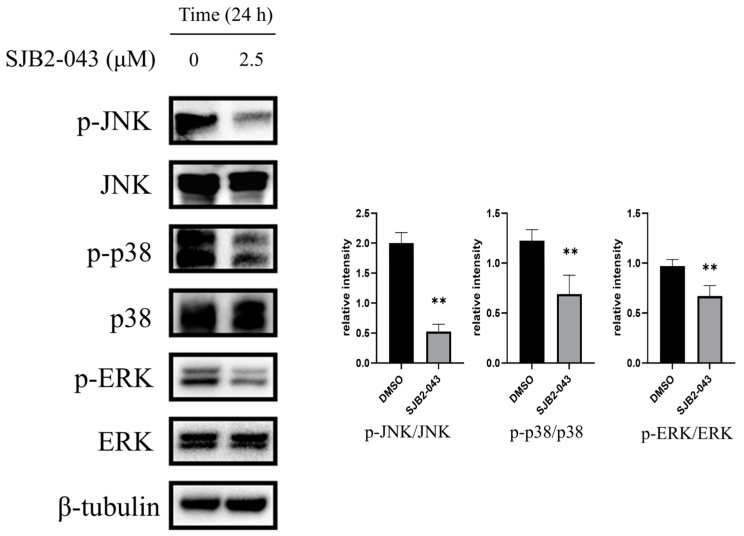
Effects of SJB2-043 on MAPK signaling pathway protein expression in A549 cells. WB was executed to evaluate MAPK signaling pathway protein level (p-JNK/JNK, p-p38/p38, p-ERK/ERK) in A549 cells exposed to SJB2-043 at a level of 2.5 µM for 24 h. β-tubulin served as a loading control. Data are denoted as mean ± SD (*n* = 4). Statistical analysis was executed utilizing Student’s *t*-test. ** *p* < 0.01, compared with the control group.

KEGG pathway analysis indicates that the antitumor effect of SJB2-043 on A549 cells is primarily mediated through the MAPK signaling pathway. To investigate the underlying mechanisms, we comprehensively assessed the expression levels of pivotal signaling molecules involved in this pathway, encompassing both phosphorylated and total forms of p38, JNK, and ERK. The MAPK signaling cascade represents a crucial regulatory network in oncogenesis, playing a pivotal role in modulating essential cellular processes such as proliferation, differentiation, survival, and migration. Notably, dysregulation of MAPK signaling is frequently observed across diverse malignancies, contributing significantly to tumor initiation and progression. This molecular pathway has consequently emerged as a promising therapeutic target in oncology. Of particular clinical relevance, pharmacological interventions targeting the ERK signaling node have demonstrated considerable therapeutic efficacy in various cancer types [28,29,30]. Our experimental data demonstrate that SJB2-043 exerts potent inhibitory effects on the activation of ERK, p38, and JNK, thereby suppressing MAPK-mediated signaling transduction and impeding the progression of NSCLC (Figure 8).

### 3.9. SJB2-043 Suppresses the Wnt/β-Catenin Pathway in A549 Cells

**Figure 9:**
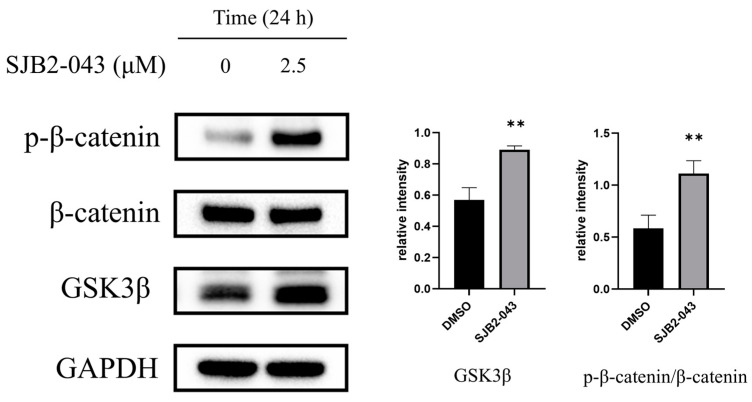
Effects of SJB2-043 on Wnt/β-catenin signaling cascade protein expression in A549 cells. WB was executed to evaluate the protein level implicated in the Wnt/β-catenin signaling cascade (GSK3β, p-β-catenin/β-catenin) in A549 cells exposed with SJB2-043 at 2.5 µM for 24 h. GAPDH served as the loading control. Data are denoted as mean ± SD (*n* = 4). Statistical significance was ascertained utilizing Student’s *t*-test. ** *p* < 0.01, compared with the control group.

The Wnt/β-catenin signaling pathway plays a pivotal role in tumor invasion and metastasis, promoting tumor progression by modulating EMT. GSK3β acts as a central regulator within this pathway. It interacts with the adenomatous polyposis coli gene, β-catenin, and axis inhibition protein (Axin) to form a degradation complex. Upon phosphorylation by GSK3β, β-catenin undergoes ubiquitination, leading to its subsequent degradation [31,32]. The expression of GSK3β can be upregulated by SJB2-043, which also enhances β-catenin phosphorylation (Figure 9). These observations suggest that SJB2-043 may inhibit NSCLC cell invasion and migration via the suppression of the Wnt/β-catenin signaling cascade.

### 3.10. SJB2-043 Inhibits the PI3K/AKT/mTOR Pathway in A549 Cells

**Figure 10:**
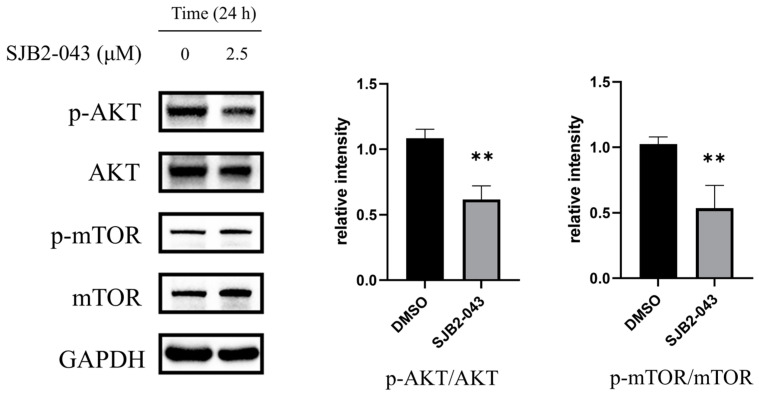
Effects of SJB2-043 on PI3K/AKT/mTOR signaling cascade protein expression in A549 cells. WB was executed to assess the protein level associated with the PI3K/AKT/mTOR signaling cascade (p-AKT/AKT, p-mTOR/mTOR) in A549 cells treated with SJB2-043 at a level of 2.5 µM for 24 h. GAPDH served as the loading control. Data are denoted as mean ± SD (*n* = 4). Statistical significance was ascertained utilizing Student’s *t*-test. ** *p* < 0.01, compared with the control group.

The PI3K/AKT/mTOR signaling pathway is frequently dysregulated in NSCLC, primarily due to various genetic and epigenetic alterations, including mutations or overexpression of EGFR [33,34]. This aberrant activation plays a pivotal role in promoting multiple oncogenic processes in NSCLC, such as tumor cell proliferation, survival, invasion, and metastasis [35]. Mechanistically, mTOR activation facilitates protein synthesis and cell proliferation, thereby accelerating NSCLC progression. Meanwhile, AKT exerts its oncogenic effects through phosphorylation of diverse downstream substrates, which subsequently regulates critical cellular processes including cell cycle progression and apoptosis [36]. KEGG pathway analysis indicates that SJB2-043 significantly impacts the PI3K/AKT/mTOR signaling pathway. To elucidate the molecular basis of SJB2-043’s anti-NSCLC properties, we examined the activated forms of AKT and mTOR, specifically p-AKT and p-mTOR. Western blot analysis revealed that, following SJB2-043 treatment, the ratios of p-AKT to total AKT and p-mTOR to total mTOR were markedly reduced in A549 cells (Figure 10). These findings suggest that SJB2-043 may inhibit NSCLC development and progression by suppressing the PI3K/AKT/mTOR signaling cascade.

## 4. Discussion

NSCLC continues to be among the primary contributors to cancer occurrence and mortality globally, presenting considerable challenges to effective clinical management. Addressing this pressing issue necessitates discovering novel therapeutic interventions and strategies. In this investigation, the effects and underlying mechanisms of the USP1 inhibitor SJB2-043 on the A549 human NSCLC cell line were investigated. The findings reveal valuable perspectives regarding the curative prospects of USP1 inhibitors in combating NSCLC.

Ubiquitin-specific proteases (USPs), key constituents of the DUB family, are integral to the regulation of biological processes, influencing pathways such as DNA damage repair, the p53 cascade, and the TGF-β signaling cascade [37,38]. In the context of tumorigenesis, USPs modulate several critical aspects of tumor biology, including cellular proliferation, apoptosis, metastasis, and regulation of the cell cycle [39]. Given their essential function in sustaining the DNA replication and repair machinery within cancer cells, USPs have been identified as promising targets for therapeutic intervention [40,41]. Notably, USP1 is frequently overexpressed in tumor cells and correlates with tumor progression, establishing it as a particularly attractive therapeutic target [42,43,44]. The findings from this investigation provide further validation for the potential of inhibiting USP1 with specific agents, such as SJB2-043, to impede the progression of NSCLC.

Tumor cell invasion and metastasis represent pivotal events in the progression and development of malignant tumors, markedly contributing to patient mortality [45]. EMT is recognized as a key mechanism in facilitating tumor cell invasion and metastasis by modifying the epithelial cell phenotype and surface antigens, thereby acquiring a mesenchymal phenotype. This transition reduces intercellular adhesion and disrupts cellular integrity, leading to a loss of order, stability, and cell polarity, resulting in a more motile and loosely organized mesenchymal-like cell phenotype [46,47]. E-cadherin, a transmembrane protein predominantly expressed in epithelial cells, is primarily localized on the lateral membranes of adjacent cells, where it serves an essential function in maintaining cell polarity and structural integrity. The reduction of E-cadherin represents a defining feature of EMT in epithelial cells, leading to mesenchymal morphological changes that enhance cell migration, invasion, and metastasis [48,49]. N-cadherin modulates EMT-associated protein levels, such as E-cadherin and vimentin, by activating the MAPK/ERK and PI3K signaling pathways, thereby promoting both EMT and tumor metastasis [45]. To counteract the tumor metastasis driven by the diminishing of cell junctions, ZO-1, a marker of epithelial cell junctions, counteracts EMT by maintaining tight junctions, preserving endothelial cell polarity, and contributing to cytoskeletal organization [50]. Abnormal expression or loss of ZO-1 is associated with various diseases, including cancer. Additionally, occludin, another critical tight junction protein, contributes to the sealing of intercellular gaps, co-polymerizes with other molecules, and regulates signaling during tight junction formation, thereby ensuring normal cellular physiological functions [51,52]. The Wnt/β-catenin signaling cascade is a well-established oncogenic pathway [53]. It has been suggested that Wnt/β-catenin signaling cascade activation in NSCLC induces EMT, promoting tumor invasion and metastasis [54]. To evaluate how SJB2-043 influences A549 cells’ metastatic capabilities, this investigation explores the effects of SJB2-043 on key EMT marker levels (E-cadherin, N-cadherin, ZO-1, occludin) and important proteins in the Wnt/β-catenin signaling cascade (GSK3β, p-β-catenin/β-catenin) through a wound-healing assay. The results reveal that the USP1 inhibitor SJB2-043 enhances E-cadherin, ZO-1, occludin, GSK3β, and p-β-catenin/β-catenin levels, while suppressing N-cadherin expression, suggesting that SJB2-043 inhibits NSCLC progression by modulating EMT and Wnt/β-catenin signaling. Furthermore, the wound-healing assay demonstrates that SJB2-043 reduces the migration capacity of A549 cells, with its inhibitory effect becoming more pronounced at higher concentrations.

Abnormal cell proliferation is a key feature of cancer cells, which arises due to the disruption of normal cell cycle regulation and irregular cell apoptosis [55]. Therefore, to investigate whether SJB2-043 inhibits cell proliferation by modulating the cell cycle and inducing apoptosis, FCM was employed to assess the impact of SJB2-043 on the cell cycle and apoptosis in A549 cells. The experimental findings show that SJB2-043 induces growth arrest in A549 cells at the G2 phase. To examine the molecular pathways behind this G2 phase arrest, WB analysis was utilized to measure protein levels implicated in G2 phase regulation, such as C-myc, CDK1, CyclinB_1_, and p53. The results demonstrate that SJB2-043 markedly downregulates C-myc, CDK1, and CyclinB_1_ while upregulating p53. Elevated levels of CDK1/CyclinB_1_ are closely linked to the progression and prognosis of LC [56]. p53, a critical tumor suppressor protein, serves as a central regulator of cell proliferation and apoptosis, capable of halting cell cycle progression and initiating apoptotic processes [57]. To confirm the apoptosis-inducing effect of SJB2-043 in A549 cells, FCM was employed to assess cell apoptosis. The data confirm that SJB2-043 promotes apoptosis in these cells. To investigate the underlying pathways through which SJB2-043 induces apoptosis, WB was executed to examine the expression of apoptotic markers Bax and Bcl-2. The mitochondrial apoptosis pathway is a key intrinsic mechanism of apoptosis, with the Bcl-2 protein family serving a pivotal function in regulating this process [58]. The downregulation of Bcl-2 levels coupled with elevated Bax expression is known to enhance tumor cell apoptosis in response to various external stimuli [59,60]. WB analysis revealed that SJB2-043 elevates the Bax level and reduces the Bcl-2 level.

The PI3K/AKT/mTOR and MAPK signaling cascades are integral to modulating cell growth, viability, and resistance to radiotherapy and chemotherapy [61,62,63,64]. This investigation revealed that SJB2-043 markedly inhibited the activation of several key proteins within these pathways, including p-AKT/AKT, p-mTOR/mTOR, p-ERK/ERK, p-p38/p38, and p-JNK/JNK. These results indicate that SJB2-043 may mediate its anti-NSCLC effects, at least in part, by modulating these critical signaling cascades.

## 5. Conclusions

**Figure 11:**
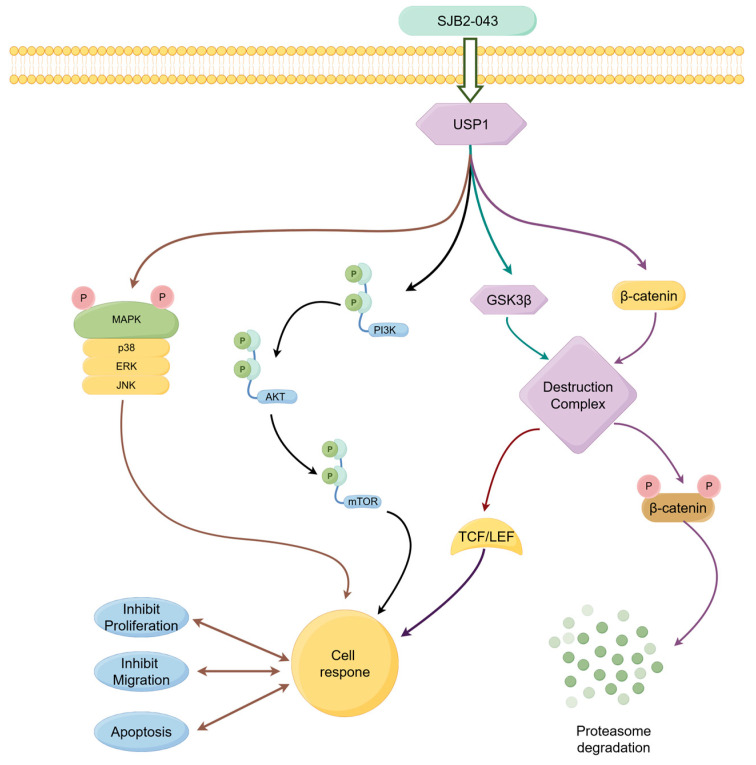
The mechanism of action of SJB2-043 in A549 cells. SJB2-043 influences cellular responses through multiple pathways (PI3K/AKT/mTOR, Wnt/β-catenin, MAPK pathways), including inhibiting proliferation, migration, as well as promoting apoptosis.

This investigation establishes USP1 as a druggable vulnerability in NSCLC through the development of SJB2-043, a mechanistically novel deubiquitinase inhibitor with potent on-target activity. RNA sequencing revealed that SJB2-043-mediated USP1 ablation induces catastrophic disruption of three evolutionarily conserved pro-survival networks: MAPK, Wnt/β-catenin, and PI3K/AKT/mTOR pathways (Figure 11). The compound’s therapeutic efficacy was validated through functional assays demonstrating dose-dependent pathway inactivation.

These findings redefine USP1’s role as a master coordinator of oncogenic plasticity in NSCLC, positioning targeted USP1 inhibition as a paradigm-shifting strategy to counter adaptive resistance mechanisms. Particularly noteworthy is SJB2-043’s ability to concurrently dismantle multiple compensatory pathways—a pharmacological advantage that may circumvent the limitations of single-pathway-targeted therapies. The robust preclinical evidence presented herein provides both a mechanistic blueprint for USP1-directed therapeutics and a translational roadmap for clinical development, including biomarker-driven patient stratification and rational combination regimens.

## Data Availability

All data associated with this study are available from the corresponding authors upon reasonable request.

## Supplementary Materials

The following supporting information can be downloaded at: https://www.mdpi.com/article/10.3390/cimb47030155/s1.

## Abbreviations

The following abbreviations are used in this manuscript:

EMT	epithelial–mesenchymal transition
NSCLC	non-small cell lung cancer
DUBs	Deubiquitinating enzymes
USP1	ubiquitin-specific peptidase 1
KEGG	Kyoto Encyclopedia of Genes and Genomes
RNA-seq	RNA-sequencing
FCM	Flow cytometry
PI	propidium iodide
PBS	phosphate buffered saline
CF	Colony formation
WB	Western blot
DEGs	differentially expressed genes
