# Supplementary material for: SJB2-043, a USP1 Inhibitor, Suppresses A549 Cell Proliferation, Migration, and EMT via Modulation of PI3K/AKT/mTOR, MAPK, and Wnt Signaling Pathways
Source: Curr Issues Mol Biol. 2025 Feb 27;47(3):155. doi: 10.3390/cimb47030155 (PMC11941171; doi:10.3390/cimb47030155)
Supplement: Supplementary file 1 [file cimb-47-00155-s001.zip › cimb-3475498-supplementary.pdf]

Supplementary Materials

Figure S1. Effect of SJB2-043 on the proliferation of Beas2B cells.

Methods

Cell Culture

Beas2B cells were purchased from ATCC and cultured in DMEM medium (Gibco, USA) supplemented with 10% fetal bovine serum (Vivacell, China) and 1% penicillin-streptomycin (Gibco, USA) at 37°C in a 5% CO2 atmosphere. The cells were passaged regularly.

Results

SJB2-043 has minimal effect on Beas2B

The results indicated that SJB2-043 had no noticeable effects on normal human lung epithelial cells (Beas2B), even at a concentration of 10 μM, and therefore exhibited low inhibitory activity against normal cells (Figure S1).

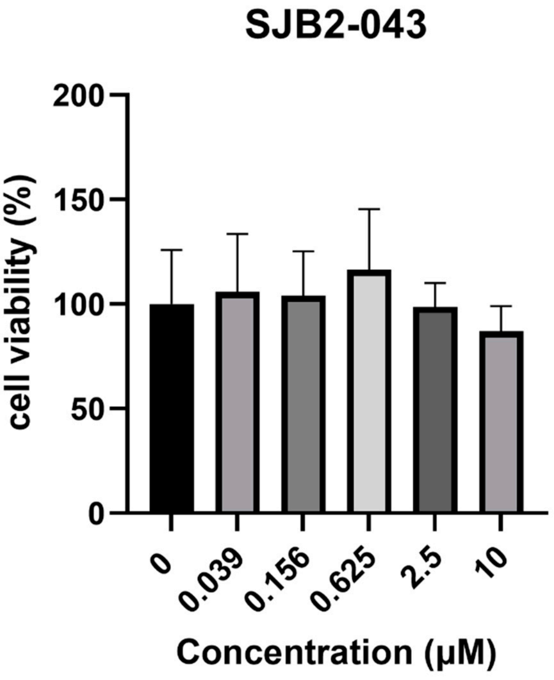

Figure S1. Effect of SJB2-043 on the proliferation of Beas2B cells.

Beas-2B cells were treated with increasing concentrations of SJB2-043 (0, 0.039, 0.156, 0.625, 2.5, 10 μM) for 24 h. Cell viability was measured using a CCK-8 assay. Data are presented as mean ± SEM (n = 6). Statistical significance was analyzed using one-way ANOVA (* $p < 0.05$, ** $p < 0.01$).
